# Editorial: The Role of Exosomes in Metabolic and Endocrine Disease

**DOI:** 10.3389/fendo.2022.859650

**Published:** 2022-03-22

**Authors:** Bei Guo, Ling-Qing Yuan

**Affiliations:** National Clinical Research Center for Metabolic Diseases, Department of Metabolism and Endocrinology, The Second Xiangya Hospital, Central South University, Changsha, China

**Keywords:** exosomes, bone, diabetes, metabolic syndrome, endocrine disease

The discovery of exosomes has a history of 36 years, but it has gradually become a research hotspot in the recent 10 years. The study of exosomes is multisystem; however, the role of exosomes in the frontier of metabolism and endocrinology has become more important since the crosstalk between cells and organs is an immensely important function of exosomes, and exosomes exert their effects in an endocrine or paracrine manner.

In the present Research Topic includes four research articles and five reviews, which focused on exosomes as messengers of intercellular or inter-organ communication in metabolic and endocrine diseases.

There are four papers that discussed the role of exosomes in bone and muscle metabolism.

The original research of Li et al., titled “*Exosomes Derived From M2 Macrophages Facilitate Osteogenesis and Reduce Adipogenesis of BMSCs*”, investigated exosomes derived from M2 macrophages that facilitate osteogenesis and decrease the adipogenesis of bone marrow mesenchymal stem cells through the miR-690/IRS-1/TAZ axis, which suggested that exosomes derived from M2 macrophages might possibly provide a therapeutic tool for bone loss diseases.

In the field of bone metabolism, the review by Yang et al., “*Exosomes: A Friend or Foe for Osteoporotic Fracture?*”, discussed the role of exosomes in the occurrence and healing of osteoporotic fractures. Bone-related cell-derived exosomes have dual function in osteoporosis. Exosomes secreted by osteoclasts, osteoblasts, and mesenchymal stem cells have bilateral effects of accelerating and slowing down osteoporosis. Exosomes derived from vascular endothelial cells mainly inhibit the process of osteoporosis. More research on the potential mechanism of myocyte- and macrophage-derived exosomes in osteoporosis is still needed to make up for the limited data available. The authors concluded the diagnostic and therapeutic applications of exosomes in osteoporotic fracture by regulating osteoblastogenesis, osteoclastogenesis, and angiogenesis.

In addition, Aoi et al., in the mini review “*Roles of Skeletal Muscle-Derived Exosomes in Organ Metabolic and Immunological Communication*”, discussed that exosomes derived from skeletal muscles can be recognized as myokines, which play their role by regulating the physiological and pathological states of the target organ, including physical fitness, muscle disorders, and lifestyle-related diseases. In their opinion, exosomes have potential application as biomarkers reflecting the metabolic and immune status.

Moreover, Li et al., in another research titled “*BMSC-Derived Exosomes Inhibit Dexamethasone-Induced Muscle Atrophy via the miR-486-5p/FoxO1 Axis*”, investigated the mechanism of bone marrow stromal cell-derived exosomes (BMSC-Exos) in suppressing dexamethasone-induced myotube atrophy and muscle wasting by delivery of miR-486-5p, which reduced the expression of forkhead box protein O1 (FoxO1) in the muscle nucleus. The authors suggested that it might be a new biotherapy for muscle atrophy based on exosomes.

Four papers focused on diabetes, metabolic syndrome, or obesity.

The original study “*Metabolic Syndrome Is Associated With Altered mRNA and miRNA Content in Human Circulating Extracellular Vesicles*” reported by Li et al. provided data on the changes in the significance of the modified cargo of circulating extracellular vesicles in human subjects with metabolic syndrome (MetS) or age-matched lean controls. The authors concluded that exosomes can potentially serve as important regulators, biomarkers, and targets in the progression and treatment of MetS.

The review “*Adipocyte–Endothelium Crosstalk in Obesity*” by Sabaratnam and Svenningsen illustrated how obesity changes the adipose tissue microenvironment through exosome-mediated communication and how this communication regulates systemic energy homeostasis in metabolic disorders. Exosomes mediate the information exchange between a variety of cell types in adipose tissue, especially between adipocytes and endothelial cells. The adiponectin secreted by adipocytes promotes endothelial cells in the whole body to secrete exosomes, which remove excess or unnecessary substances in endothelial cells and maintain cell homeostasis.

The review of Hu et al., “*Clinical Translational Potentials of Stem Cell-Derived Extracellular Vesicles in Type 1 Diabetes*”, summarized the therapeutic potential of stem cell-derived extracellular vesicles for the treatment of type 1 diabetes (T1D) by modulating the immune response and overcoming the deficit of islet β cells. The authors illustrated that the diversity and low yield of production of native extracellular vesicles, as well as their short half-life and the off-target effects of their functions post-administration, have limited their application, while bioengineered extracellular vesicles can facilitate the clinical translation of extracellular vesicles for the treatment of T1D.

The review by Mattke et al., “*Role of Exosomes in Islet Transplantation*”, discussed the potential of exosomes as biomarkers of islet stress and injury, inflammation or immune response, and/or as therapeutic methods for clinical islet transplantation. More and more miRNAs are identified in exosomes collected during islet isolation, which can be used as biomarkers for islet stress and injury, inflammation, or immune response. On the other hand, therapy of mesenchymal stem cell-derived exosomes has the potential to increase the vitality and function of isolated and transplanted islets.

Moreover, one paper revealed the diagnostic function of exosomes on 11β-HSD2-related hypertension.

As the relevance of exosomal cargo alterations has been confirmed in the pathology of numerous metabolic and endocrine diseases, the use of messenger RNA (mRNA) levels in exosomes as a diagnostic tool has gained interest. De Santis et al., in “*Detection of Urinary Exosomal HSD11B2 mRNA Expression: A Useful Novel Tool for the Diagnostic Approach of Dysfunctional 11β-HSD2-Related Hypertension*”, investigated the relationship between urinary exosomal HSD11B2 and hypertension status, aiming to identify an accurate method to assess HSD11B2 mRNA from urinary exosomes in samples from family members affected by apparent mineralocorticoid excess and in patients with low renin essential hypertension. The authors provided evidence that the detection of mRNA in exosomes is of great significance for the diagnosis of hypertension with 11β-HSD2 imbalance.

Therefore, the special issue summarizes the current knowledge and focuses on new insights into the role of exosomes in metabolic and endocrine diseases.

## Author Contributions

All authors listed have made a substantial, direct, and intellectual contribution to the work and approved it for publication.

## Funding

This work was supported by funding from the National Natural Science Foundation of China (nos. 81770881 and 82070910), the Key R&D Plan of Hunan Province (2020SK2078), and The Fundamental Research Funds for Central Universities of Central South University (2021zzts0387).

## Conflict of Interest

The authors declare that the research was conducted in the absence of any commercial or financial relationships that could be construed as a potential conflict of interest.

## Publisher’s Note

All claims expressed in this article are solely those of the authors and do not necessarily represent those of their affiliated organizations, or those of the publisher, the editors and the reviewers. Any product that may be evaluated in this article, or claim that may be made by its manufacturer, is not guaranteed or endorsed by the publisher.

